# Analysis of Socioeconomic Status in the Patients with Rheumatoid Arthritis

**DOI:** 10.3390/ijerph15061194

**Published:** 2018-06-07

**Authors:** Deng-Ho Yang, Jing-Yang Huang, Jeng-Yuan Chiou, James Cheng-Chung Wei

**Affiliations:** 1 Division of Rheumatology/Immunology/Allergy, Department of Internal Medicine, Taichung Armed-Forces General Hospital, Taichung 411, Taiwan; deng6263@ms71.hinet.net; 2 Department of Laboratory, Taichung Armed Forces General Hospital, Taichung 411, Taiwan; 3 Department of Medical Laboratory Science and Biotechnology, Central Taiwan University of Science and Technology, Taichung 406, Taiwan; 4 Division of Rheumatology/Immunology/Allergy, Department of Internal Medicine, Tri-Service General Hospital, National Defense Medical Center, Taipei 114, Taiwan; 5 Department of Medical Research, Chung Shan Medical University, Taichung 408, Taiwan; wchinyang@gmail.com; 6 School of Health Policy and Management, Chung Shan Medical University Hospital, Taichung 408, Taiwan; drchiou@hotmail.com; 7 Division of Allergy, Immunology and Rheumatology, Chung Shan Medical University Hospital, Taichung 408, Taiwan; 8 Institute of Medicine, Chung Shan Medical University, Taichung 408, Taiwan; 9 Graduate Institute of Integrated Medicine, China Medical University, Taichung 408, Taiwan

**Keywords:** rheumatoid arthritis, socioeconomic, incidence, prevalence, Taiwan

## Abstract

Rheumatoid arthritis (RA) is a systemic inflammatory disease with different etiologies in different areas. Our study focused on the prevalence of RA in Taiwan from 2001 to 2011. This study contained longitudinal enrollment files, claims data, catastrophic illness files, and treatment registries from Taiwan Longitudinal Health Insurance Research Database. We identified RA patients by ICD-9-CM code 714.0. The demographical variables including age, sex, income and area of registration were evaluated. The multivariate Poisson regression was applied to calculate relative risk for developing RA. In Taiwan, the ratio of female to male was about 5:1. From 2001 to 2011, significant increasing prevalence of RA, from 0.07% to 0.14%, was found in women. The prevalence of RA was increasing 6% per year in both sex groups. The annual incidence rate (per 10,000 person years) ranged from 1.62 to 2.02 (female: 2.30–3.14; male: 0.71–1.17) from 2003 to 2011. City area had lowest incidence rate of RA compared with suburban or rural area. Higher incidence of RA was observed among lower socioeconomic status. The prevalence of RA was rising from 0.07% in 2001 to 0.14% in 2011. Incidence was about 2/10,000 person-years and female to male ratio was 5:1. Lower socioeconomic status and living rural region might be a risk factor for developing RA.

## 1. Introduction

Rheumatoid arthritis (RA) is a systemic inflammatory disease with polyarthritis over bilateral hands, wrists, elbows, knees, ankles and feet. At the same time, elevation of circulating biomarkers of ESR and CRP with positive rheumatoid factor or anti-CCP autoantibodies is also observed. From the previous reports, the disease prevalence of RA is about 0.3–6% in general population with predominance of women [1]. Different prevalence and incidence of RA with large variation are observed for different ethnicities or areas. Higher prevalence seems to be found in indigenous population of North American (about 6.8%) [2]. Lower prevalence of RA is found in France (about 0.31%) [3]. Women are 2–3 times more likely to develop RA than men. RA may occur at any age, but the peak incidence is between the ages of 40 and 60. The possible causes of RA include gender, genetic susceptibility, cigarette smoking, shared epitope and infections. Hormonal, genetic, environmental and infection all play important roles in the susceptibility or progression of RA. Other risk factors related RA include occupational exposure, low to moderate alcohol consumption, obesity, duration of breastfeeding, and socioeconomic status [4,5,6,7]. Different occupation with silica exposure might be associated with increased risk of developing RA [8]. Some studies demonstrate the relation of RA and chronic periodontal inflammation or gut microbiome [9,10,11]. Different diet and nutritional status may influence the gut microbial communities and change the balance of innate/adaptive immune system [10,12]. Posttraumatic stress disorder, stress related physical disorders and atopic dermatitis may also increase the risk of developing RA [4]. The different educational levels may influence the presentation of disease activity and functional disability of RA patients [13]. The development and disease progression of RA may be influenced by both genetic and environmental factors. Adequate evaluation and understanding of numerous environmental risk factors may be another aim of preventing the progression of RA. Therefore, our study was focused on the prevalence of RA in the population of Taiwan and evaluating the risk factors including gender, age, socioeconomic level and living district. 

## 2. Materials and Methods

### 2.1. Data Source

Taiwan National Health Insurance (NHI) covered about 98% residents in 2005. The Taiwan Longitudinal Health Insurance Research Database (LHIRD) involved 1 million beneficiaries who were randomly sampled from NHI enrollment list in 2005. The LHIRD constructed by National Health Research Institutes, and these datasets were broadly used in academic studies [14,15]. This study used 2005 LHIRD that contained longitudinal enrollment files, claims data, catastrophic illness files, and treatment registries from 2001 to 2011. This study was approved by the Institutional Review Board of Chung Shan Medical University Hospital (Code No.: CS15134), and the 2005 LHIRD was an encrypted and anonymous secondary database.

### 2.2. Annual Denominator Data

Population data were identified from enrollment files, and the demographical variables including age, sex, income and area of registration were selected. The economic status was estimated by their insurable monthly incomes, and classified into three groups (<1000, 1000–2000 and ≥2000 US dollars) by the exchange rate (about 1 USD = 30 NTD). The definition of urbanization levels was modified from the method mentioned by Liu et al. [16]. Originally, level of urbanization was seven categories according to demographic components in communities. In this study, urbanization was regrouped into three levels (City, Township, and Rural), as in a previous study [17]. The prevalence or incidence rate became less representative among younger or elder group, thus we excluded observations for those who were <10 or ≥80 years old from the annual population, and age groups were divided into seven groups with 10-year intervals.

### 2.3. Selection of RA Case

The Catastrophic illness files were used to identify the RA patients. National Health Insurance Administration reviewed the application of catastrophic illness certification, and the ineligible patients were rejected after assessment. We identified the RA patient by International Classification of Diseases, 9th Revision, Clinical Modification, ICD-9-CM code 714.0. The annual prevalent cases were regarded as patients with catastrophic illness certification and at least 1 RA treatment in the calendar year. The annual new cases were collected from 2003 to 2011 by excluding the prevalent cases from 2001 to 2002.

### 2.4. Statistical Analyses

All statistical analyses were conducted using the SAS statistical package (Version 9.4; SAS Institute, Inc., Cary, NC, USA). Demographic-specific annual prevalence (2001–2011) or incidence (2003–2011) of RA was calculated by dividing amount of cases by population denominator. The Poisson distribution was assumed for estimating 95% confidence intervals (CI). The trends of prevalence or incidence rate were plotted stratified by gender and age group. 

Poisson regression was conducted to analyze linear trend of rates. The multivariate Poisson regression was applied to calculate relative risk (RR, and 95% Confidence Interval, 95% CI) of demographic variables on RA incidence while adjusting other covariates. Two-sided *p*-value < 0.05 was considered statistically significant.

## 3. Results

### 3.1. Increasing Prevalence of RA in Taiwan from 2001 to 2011

Table 1 shows increasing prevalence (per 10,000 people) of RA in people aged 10–70 years old, from 6.63 (95% CI: 6.08–7.23) in 2001 to 14.01 (95% CI: 13.24–14.82) in 2011. The prevalence of RA was double in 2011. Furthermore, sex stratified prevalence was also calculated, finding that female prevalence was 10.55 (95% CI: 9.58–11.62) in 2001 and 22.25 (95% CI: 20.90–23.68) in 2011, while male prevalence was 2.64 (95% CI: 2.18–3.21) in 2001 and 5.37 (95% CI: 4.71–6.12) in 2011. The incidence of RA was women predominant. The ratio of women to men was about 5:1. From 2001 to 2011, significant increasing prevalence of RA was found in the women group (Table 1). 

### 3.2. Different Prevalence of RA in Different Age, and Higher Prevalence in the Group of 70–79 Years Old Especially

**Figure 1:**
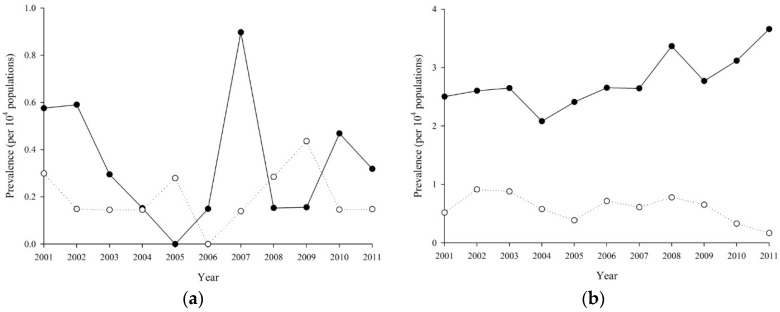

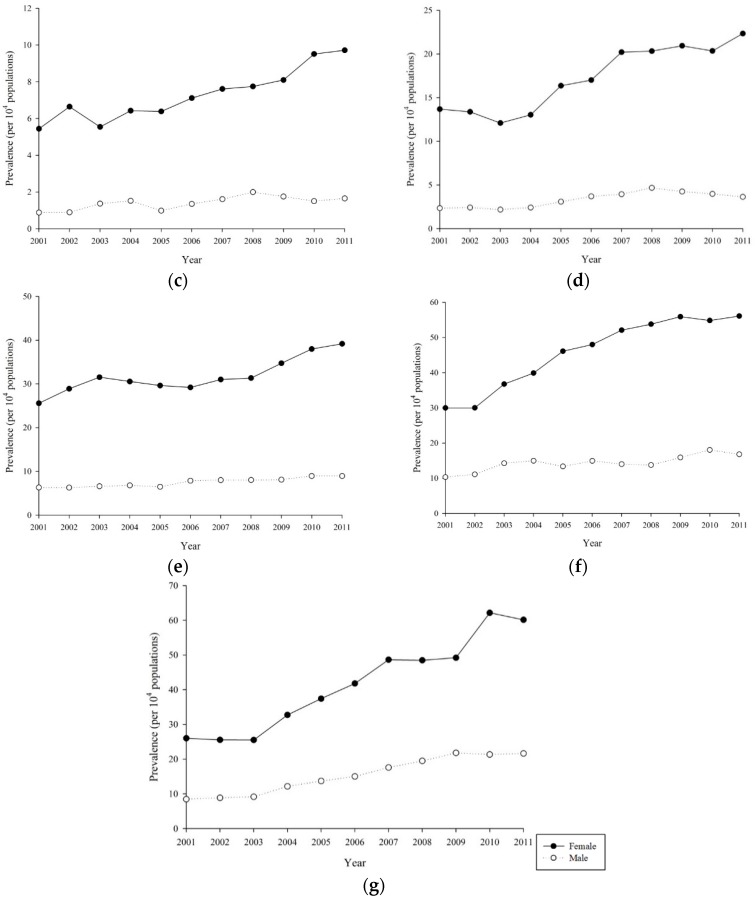
Prevalence of Rheumatoid Arthritis from 2001 to 2011 by age groups: (**a**) 10–19 years old; (**b**) 20–29 years old; (**c**) 30–39 years old; (**d**) 40–49 years old; (**e**) 50–59 years old; (**f**) 60–69 years old; and (**g**) 70–79 years old.

Age-sex stratified prevalence of RA is reported in Figure 1a–g. For female, the exponential coefficients of calendar year (per year) were 0.96 (*p* = 0.572), 1.04 (*p* = 0.081), 1.06 (*p* ≤ 0.001), 1.06 (*p* ≤ 0.001), 1.04 (*p* ≤ 0.001), 1.06 (*p* ≤ 0.001) and 1.10 (*p* ≤ 0.001) at 10–19, 20–29, 30–39, 40–49, 50–59, 60–69 and 70–79 years old, respectively; for male, those were 1.01 (*p* = 0.883), 0.94 (*p* = 0.213), 1.06 (*p* = 0.042), 1.07 (*p* ≤ 0.001), 1.04 (*p* = 0.008), 1.04 (*p* = 0.005) and 1.11 (*p* ≤ 0.001), respectively. We also tested the interactions between age group and calendar year; the interaction term was not significant for female (*p* = 0.389) or male (*p* = 0.071). Higher prevalence of RA was found in patients with age 48–63. From 2001 to 2011, progressive increasing prevalence of RA was observed in all patients. However, significant increasing prevalence was found after 30 years old. Higher prevalence of RA was found in the group of 70–79 years old. Overall, the increasing prevalence of RA was women predominant with elder age. 

### 3.3. Higher Prevalence of RA in the People with Women, Older Age, Living in Rural Area or Low-Income Earner (Monthly Income Less Than 1000 USD)

After using multiple Poisson regression, the prevalence of RA was progressively increasing every year. The trend of RA prevalence was evaluated in Table 2, and the prevalence of RA was increasing 6% per year in both sex groups from 2001 to 2011. Gender factors affected susceptibility to RA. Women were three to four times more likely to develop RA than men (RR: 3.77, 95% CI: 3.58–3.96). The prevalence of RA was significantly increased after the age of 40. We also evaluated the different socioeconomic status and living district in the patients with RA. Living in rural area may increase the risk of RA when compared with living urban area. Lower income earner (monthly income less than 1000 USD) was also associated with increased the risk of RA. 

### 3.4. Lower Socioeconomic Status, Women, and Living Closed Rural Area Increased the Risk of RA

The incidence rates of RA are shown in Table 3. The annual incidence rate (per 10,000 person years) ranged from 1.62 to 2.02 (female: 2.30 to 3.14; male: 0.71 to 1.17) from 2003 to 2011. The incidence rate was increasing by age, the rate was 0.14 and 5.49 (female: 0.19 and 7.74; male: 0.10 and 3.20) in the youngest (10–19 years old) and eldest (70–79 years old) groups, respectively. City areas had lowest incidence rate of RA compared with suburban or rural areas. When people lived in rural areas, higher incidence of RA was found, and the finding especially significantly higher in the women. The highest income level had lowest rate: 1.36 per 10,000 person years. Higher incidence of RA was observed among lower socioeconomic status when monthly income was less than 1000 USD. 

### 3.5. The Incidence of RA was Increasing with Age Except Women or Men

**Figure 2:**
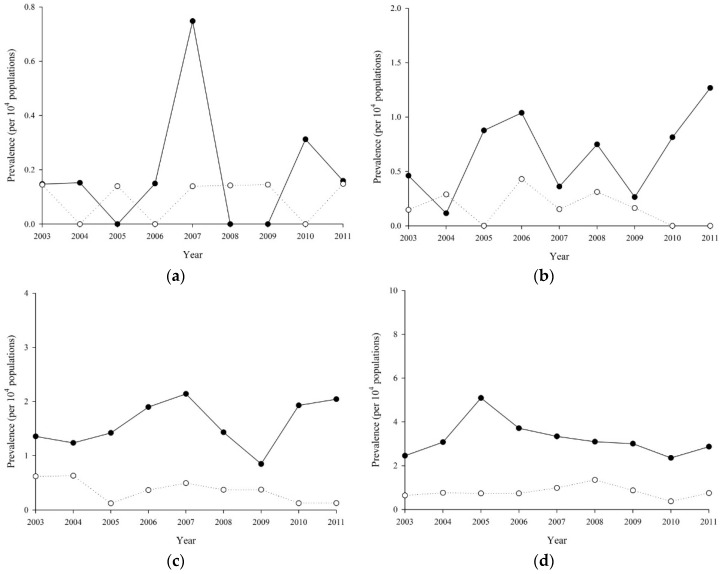

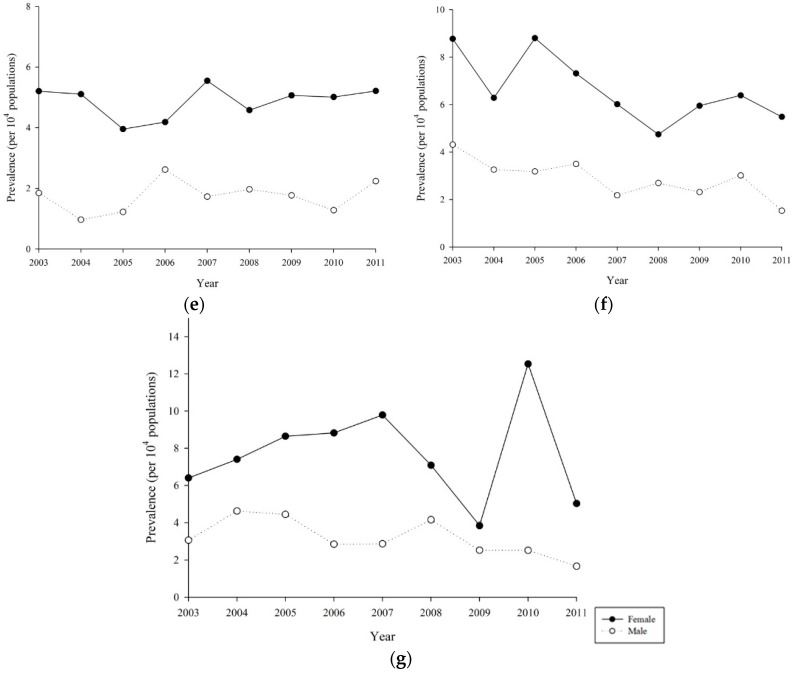
Incidence rate of Rheumatoid Arthritis from 2001 to 2011 by age groups: (**a**) 10–19 years old; (**b**) 20–29 years old; (**c**) 30–39 years old; (**d**) 40–49 years old; (**e**) 50–59 years old; (**f**) 60–69 years old; and (**g**) 70–79 years old.

Age-sex stratified incidence rate is plotted in Figure 2a–g. For female, the exponential coefficient of period (per year) effects were 0.90 (*p* = 0.196), 0.94 (*p* = 0.115), 0.91 (*p* ≤ 0.001), 0.88 (*p* ≤ 0.001), 0.88 (*p* ≤ 0.001), 0.87 (*p* ≤ 0.001) and 0.93 (*p* ≤ 0.001) at 10–19, 20–29, 30–39, 40–49, 50–59, 60–69 and 70–79 years old, respectively; for male, those were 0.96 (*p* = 0.726), 0.83 (*p* = 0.028), 0.87 (*p* = 0.012), 0.93 (*p* = 0.036), 0.93 (*p* = 0.009), 0.87 (*p* ≤ 0.001), and 0.91 (*p* = 0.004), respectively. The interaction between age and period effect on RA incidence was not significant for female (*p* = 0.346) or male (*p* = 0.846).

### 3.6. Elder and Women were the Risk for Developing RA

Relative risks for demographic factors on RA incidence are listed in Table 4. After adjusting for sex, age groups, urbanization and income levels, the period effect was not significant with exp(b) = 0.99 and *p* = 0.175 (for female *p* = 0.613; for male *p* = 0.0607). Female had 2.90 times (95% CI 2.57–3.27) risk when compared with male. The increasing RR was observed in elder group, and it was 38.55 (95% CI 23.57–63.07) in 70–79 years old compared with 10–19 years old people. The trend of income effect was significant in male subgroup (*p* = 0.0304), but not in female (*p* = 0.5631). 

## 4. Discussion

In our study, the prevalence of RA was increasing in Taiwan from 2001 to 2011 (from 6.63/10,000 to 14.01/10,000). The incidence of RA was women predominant and was increasing with age except when separated by sex. Lower socioeconomic status or living closed rural area might serve as a risk for developing RA. 

Our study showed that age and gender play important roles in developing RA. From previous studies, the peak onset of RA is between the age of 40 and 60. However, our study showed that the incidence of RA increased in women after the age of 40 (40–49: RR = 2.01; 50–59: RR = 3.34; 60–69: RR = 4.79; 70–79: RR = 5.49) and the peak of onset was between the age of 70 and 79 (RR: 5.49, 95% CI: 4.83–6.23, Table 3). Among the women after age of 40, the diagnosis of RA should always be kept in mind due to high peak incidence of RA from our study. Women were four times more likely to develop RA than men (Table 1). The prevalence and incidence of RA was 14.01/10,000 (0.1401%) and 1.85/10,000 person-years, respectively, in 2011. The annul incidence of RA was progressive increasing between 2003 and 2011. Overall, the prevalence of RA also increased from 0.063% to 0.1401% between 2003 and 2011 in Taiwan. The prevalence of disease is associated with the incidence, disease duration and mortality. In Taiwan, the strong increase in the prevalence of RA may be due to adequate and timely immunotherapy under National Health Insurance. Patients with RA receive immediate or early diagnosis by rheumatologist. Significant elevation of incidence was found. Therefore, the prevalence of RA was increasing under good and convenient health care access for past years. The prevalence of RA has large variation for different ethnicities or areas. In Taiwan, the prevalence of RA is relatively low when compared other areas [2,3]. This condition might be because early RA patients with polyarthritis do not fulfil the old ACR criteria of 1988. We used the ICD 9: 714.0 to confirm the diagnosis of RA from the Taiwan LHIRD. Early RA patients who do not fulfill RA classification criteria were usually missed by the clinical doctors due to seronegative RA or presentation of monoarthritis. Therefore, the diagnosis rate of RA was lower than other areas. However, the incidence of RA is decreasing with a reduction in prevalence in recent years from the recent study of UK [18]. The incidence of RA is 3.81/10,000 person-years and the prevalence is 0.67% in UK. Lower incidence was found in Taiwan (1.85/10,000 person-years in Taiwan vs. 3.81/10,000 person-years in UK). The development of RA is associated with genetic and nongenetic pathogenesis. The development of RA is associated with genetic and nongenetic factors. The genetic predisposition may be the major role for developing RA [19]. 

Lower socioeconomic status, as measured by housing-based index (summed z-score for housing value, square footage and number of bedrooms and bathrooms), is associated with the risk of developing RA with higher mortality rate [20]. In our study, socioeconomic status was measured by personal monthly income. Lower socioeconomic status was defined as monthly income less than 1000 USD. Higher incidence rate was observed in females living in rural areas when compared with those living in urban areas (3.65/10,000 person-years vs. 2.58/10,000 person-years, Table 3). From the previous study, patients with higher educational background had less pain and less functional disability [13]. The childhood socioeconomic status of RA patients, such as participant homeownership or parental education, also influenced the disease outcome and progression [21]. The socioeconomic status with influencing the RA disease activity or risk includes educational level, occupation, income, family member and living areas [22]. Lower socioeconomic status may be associated with smoking, obesity, poor nutrition and higher frequency of chronic disease. The exposure of cigarette smoking is a strong risk factor in the pathogenesis of developing RA with adequate genetic background [23]. Smoking may induce the production of cirullinated proteins with abnormal development of multiple circulating autoantibodies including RF and anti-CCP autoantibodies [24]. Ingestion of high salt and low fibrate diet related to overweight or obesity may also play a major role in the development of RA [25]. Delayed diagnosis was usually found in RA patients with lower income. Higher education and higher income were factors associated with early diagnosis of RA [26]. Anti-CCP antibody positivity and low income represent severe risks for increasing time to diagnosis [27]. The predictors of higher fear-avoidance for RA patients include high activity limitation, male sex, income below average and elevated levels of anxiety/depression from a longitudinal study [28]. Therefore, the socioeconomic status may be associated with the adequate receiving therapy and follow up in the patients with RA.

There were several limitations in the present study: Early RA patients who do not fulfill RA classification criteria were usually missed by the clinical doctors due to seronegative RA or presentation of monoarthritis. Data from Taiwan LHIRD may lose RA patients without regular follow up. RA patients who are evading taxation were a bias to evaluate the socioeconomic status. 

## 5. Conclusions

In summary, various environmental factors may serve as important components influencing and modifying the risk for disease progression in addition to genetic background. In Taiwan, the prevalence of RA was about 0.15% with higher prevalence in female (5:1). The annual incidence of RA was about 2/10,000 person-years. Lower socioeconomic status (e.g., lower monthly income or living in rural regions) might be a risk factor for developing RA.

## Figures and Tables

**Table 1:** Prevalence (per 10,000 people) of Rheumatoid Arthritis among 10–79 years old people in Taiwan from 2001 to 2011.

Year	All			Female			Male		
	Population	Case	Prevalence (95% CI)	Population	Case	Prevalence (95% CI)	Population	Case	Prevalence (95% CI)
2001	778,103	516	6.63 (6.08–7.23)	392,460	414	10.55 (9.58–11.62)	385,643	102	2.64 (2.18–3.21)
2002	789,642	565	7.16 (6.59–7.77)	397,925	453	11.38 (10.38–12.48)	391,717	112	2.86 (2.38–3.44)
2003	814,451	627	7.70 (7.12–8.33)	417,117	496	11.89 (10.89–12.99)	397,334	131	3.30 (2.78–3.91)
2004	821,388	686	8.35 (7.75–9.00)	419,710	540	12.87 (11.83–14.00)	401,678	146	3.63 (3.09–4.27)
2005	864,560	777	8.99 (8.38–9.64)	439,144	626	14.26 (13.18–15.42)	425,416	151	3.55 (3.03–4.16)
2006	860,999	847	9.84 (9.20–10.52)	439,640	669	15.22 (14.11–16.41)	421,359	178	4.22 (3.65–4.89)
2007	859,730	950	11.05 (10.37–11.78)	440,212	761	17.29 (16.10–18.56)	419,518	189	4.51 (3.91–5.20)
2008	859,891	996	11.58 (10.89–12.33)	440,202	789	17.92 (16.72–19.22)	419,689	207	4.93 (4.30–5.65)
2009	851,996	1052	12.35 (11.62–13.12)	435,757	835	19.16 (17.91–20.51)	416,239	217	5.21 (4.56–5.96)
2010	861,288	1157	13.43 (12.68–14.23)	440,785	930	21.10 (19.79–22.50)	420,503	227	5.40 (4.74–6.15)
2011	862,136	1208	14.01 (13.24–14.82)	441,393	982	22.25 (20.90–23.68)	420,743	226	5.37 (4.71–6.12)

**Table 2:** Relative risk of prevalent cases of Rheumatoid Arthritis in Taiwan from 2001 to 2011 by using multiple Poisson regression *.

	All		Female		Male	
	RR (95% CI)	*p* Value	RR (95% CI)	*p* Value	RR (95% CI)	*p* Value
Year (per 1 year)	1.06 (1.05–1.07)	<0.0001	1.06 (1.05–1.07)	<0.0001	1.06 (1.05–1.08)	<0.0001
Sex (ref: Male)						
Female	3.77 (3.58–3.96)	<0.0001	-	-		
Age(ref: 10–19 years old)						
20–29	6.34 (4.56–8.83)	<0.0001	8.08 (5.35–12.19)	<0.0001	3.28 (1.83–5.89)	<0.0001
30–39	16.61 (12.08–22.83)	<0.0001	21.37 (14.33–31.86)	<0.0001	8.24 (4.81–14.10)	<0.0001
40–49	39.22 (28.67–53.68)	<0.0001	50.42 (33.95–74.88)	<0.0001	19.57 (11.63–32.93)	<0.0001
50–59	73.71 (53.93–100.77)	<0.0001	91.8 (61.87–136.20)	<0.0001	43.29 (25.87–72.44)	<0.0001
60–69	110.97 (81.18–151.7)	<0.0001	133.47 (89.95–198.09)	<0.0001	74.90 (44.81–125.16)	<0.0001
70–79	105.67 (77.19–144.65)	<0.0001	123.24 (82.91–183.19)	<0.0001	77.08 (46.03–129.08)	<0.0001
Residential urbanization (ref: Urban)						
Sub-urban	1.05 (1.00–1.09)	0.0518	1.01 (0.96–1.06)	0.7885	1.24 (1.13–1.37)	<0.0001
Rural	1.00 (0.93–1.07)	0.9227	0.92 (0.85–1.00)	0.0562	1.31 (1.14–1.50)	0.0002
Income (ref: <30,000)						
30,000–60,000	0.88 (0.83–0.94)	0.0001	0.94 (0.88–1.01)	0.1041	0.72 (0.63–0.83)	<0.0001
≥60,000	0.79 (0.70–0.89)	0.0001	0.99 (0.85–1.14)	0.8695	0.54 (0.43–0.68)	<0.0001

* Models was adjusted for calendar year, sex, age groups, urbanization and income levels.

**Table 3:** Incidence rate (per 10,000 person years) of Rheumatoid Arthritis among 16–79-year-old people in Taiwan from 2003 to 2011 by specific sub-groups.

	All			Female			Male		
	Py *	New Case	Incidence Rate * (95% CI)	Py *	New Case	Incidence Rate * (95% CI)	Py *	New Case	Incidence Rate * (95% CI)
Year									
2003	813,767	144	1.77 (1.50–2.08)	416,574	103	2.47 (2.04–3.00)	397,193	41	1.03 (0.76–1.4)
2004	820,579	139	1.69 (1.43–2.00)	419,077	100	2.39 (1.96–2.90)	401,502	39	0.97 (0.71–1.33)
2005	863,607	170	1.97 (1.69–2.29)	438,407	134	3.06 (2.58–3.62)	425,200	36	0.85 (0.61–1.17)
2006	859,912	174	2.02 (1.74–2.35)	438,793	128	2.92 (2.45–3.47)	421,119	46	1.09 (0.82–1.46)
2007	858,510	173	2.02 (1.74–2.34)	439,262	134	3.05 (2.58–3.61)	419,248	39	0.93 (0.68–1.27)
2008	858,550	158	1.84 (1.57–2.15)	439,152	109	2.48 (2.06–2.99)	419,398	49	1.17 (0.88–1.55)
2009	850,566	138	1.62 (1.37–1.92)	434,646	100	2.30 (1.89–2.80)	415,920	38	0.91 (0.66–1.26)
2010	859,762	168	1.95 (1.68–2.27)	439,594	138	3.14 (2.66–3.71)	420,168	30	0.71 (0.50–1.02)
2011	860,498	159	1.85 (1.58–2.16)	440,107	125	2.84 (2.38–3.38)	420,391	34	0.81 (0.58–1.13)
Age									
16–19	1,218,345	17	0.14 (0.09–0.22)	590,245	11	0.19 (0.10–0.34)	628,100	6	0.10 (0.04–0.21)
20–29	1,331,445	58	0.44 (0.34–0.56)	735,024	48	0.65 (0.49–0.87)	596,421	10	0.17 (0.09–0.31)
30–39	1,469,061	145	0.99 (0.84–1.16)	747,384	119	1.59 (1.33–1.91)	721,677	26	0.36 (0.25–0.53)
40–49	1,441,546	290	2.01 (1.79–2.26)	720,385	232	3.22 (2.83–3.66)	721,161	58	0.80 (0.62–1.04)
50–59	1,127,002	376	3.34 (3.02–3.69)	569,415	278	4.88 (4.34–5.49)	557,587	98	1.76 (1.44–2.14)
60–69	622,731	298	4.79 (4.27–5.36)	323,433	213	6.59 (5.76–7.53)	299,298	85	2.84 (2.30–3.51)
70–79	435,621	239	5.49 (4.83–6.23)	219,726	170	7.74 (6.66–8.99)	215,895	69	3.20 (2.52–4.05)
Urbanization									
Urban	4,745,237	843	1.78 (1.66–1.90)	2,480,107	640	2.58 (2.39–2.79)	2,265,130	203	0.90 (0.78–1.03)
Sub-urban	2,267,357	436	1.92 (1.75–2.11)	1,113,034	317	2.85 (2.55–3.18)	1,154,323	119	1.03 (0.86–1.23)
Rural	633,157	144	2.27 (1.93–2.68)	312,471	114	3.65 (3.04–4.38)	320,686	30	0.94 (0.65–1.34)
Income									
<30,000	6,067,440	1185	1.95 (1.84–2.07)	3,253,211	902	2.77 (2.6–2.96)	281,4229	283	1.01 (0.9–1.13)
30,000–60,000	1,246,777	193	1.55 (1.34–1.78)	559,822	142	2.54 (2.15–2.99)	686,955	51	0.74 (0.56–0.98)
≥60,000	331,534	45	1.36 (1.01–1.82)	92,579	27	2.92 (2.00–4.25)	238,955	18	0.75 (0.47–1.2)

**Table 4:** Relative risk of new cases of Rheumatoid Arthritis in Taiwan from 2003 to 2011 by using multiple Poisson regression *.

	All		Female		Male	
	RR (95% CI)	*p* Value	RR (95% CI)	*p* Value	RR (95% CI)	*p* Value
Year (per 1 year)	0.99 (0.97–1.01)	0.1752	0.99 (0.97–1.02)	0.613	0.96 (0.92–1)	0.0607
Sex (ref: Male)						
Female	2.9 (2.57–3.27)	<0.0001	-	-	-	-
Age(ref: 16–19 years old)						
20–29	2.98 (1.73–5.11)	<0.0001	3.55 (1.84–6.84)	0.0002	1.8 (0.65–4.96)	0.2552
30–39	7.19 (4.34–11.9)	<0.0001	8.73 (4.7–16.22)	<0.0001	4.16 (1.7–10.17)	0.0017
40–49	14.79 (9.05–24.15)	<0.0001	17.63 (9.62–32.33)	<0.0001	9.36 (4.02–21.8)	<0.0001
50–59	24.43 (15.01–39.77)	<0.0001	26.76 (14.63–48.95)	<0.0001	20.61 (9–47.21)	<0.0001
60–69	33.61 (20.61–54.8)	<0.0001	35.41 (19.31–64.92)	<0.0001	31.44 (13.73–72)	<0.0001
70–79	38.55 (23.57–63.07)	<0.0001	41.06 (22.31–75.58)	<0.0001	34.18 (14.83–78.74)	<0.0001
Residential urbanization (ref: Urban)						
Sub-urban	1.07 (0.96–1.21)	0.2293	1.08 (0.94–1.23)	0.2867	1.08 (0.86–1.35)	0.5114
Rural	1.04 (0.87–1.24)	0.6728	1.15 (0.94–1.41)	0.1682	0.76 (0.52–1.12)	0.1698
Income (ref: ≥30,000)						
30,000–60,000	0.90 (0.77–1.06)	0.2004	0.95 (0.79–1.14)	0.5974	0.79 (0.58–1.08)	0.1453
≥60,000	0.79 (0.59–1.08)	0.1362	0.94 (0.64–1.38)	0.7465	0.64 (0.39–1.04)	0.0711

* Models was adjusted for calendar year, sex, age groups, urbanization and income levels.
